# Design and Fabrication of a Kirigami-Inspired Electrothermal MEMS Scanner with Large Displacement

**DOI:** 10.3390/mi11040362

**Published:** 2020-03-30

**Authors:** Masaaki Hashimoto, Yoshihiro Taguchi

**Affiliations:** 1 School of Integrated Design Engineering, Keio University, 3-14-1 Hiyoshi, Yokohama, Japan; hashimoto@naga.sd.keio.ac.jp; 2 Research Fellow of Japan Society for the Promotion of Science, 5-3-1 Kojimachi, Tokyo, Japan; 3 Department of System Design Engineering, Keio University, 3-14-1 Hiyoshi, Yokohama, Japan

**Keywords:** electrothermal scanner, kirigami film, large displacement, microelectromechanical system (MEMS)

## Abstract

Large-displacement microelectromechanical system (MEMS) scanners are in high demand for a wide variety of optical applications. Kirigami, a traditional Japanese art of paper cutting and folding, is a promising engineering method for creating out-of-plane structures. This paper explores the feasibility and potential of a kirigami-inspired electrothermal MEMS scanner, which achieves large vertical displacement by out-of-plane film actuation. The proposed scanner is composed of film materials suitable for electrothermal self-reconfigurable folding and unfolding, and microscale film cuttings are strategically placed to generate large displacement. The freestanding electrothermal kirigami film with a 2 mm diameter and high fill factor is completely fabricated by careful stress control in the MEMS process. A 200 μm vertical displacement with 131 mW and a 20 Hz responsive frequency is experimentally demonstrated as a unique function of electrothermal kirigami film. The proposed design, fabrication process, and experimental test validate the proposed scanner’s feasibility and potential for large-displacement scanning with a high fill factor.

## 1. Introduction

Microelectromechanical system (MEMS) scanners with large vertical actuation of both the micromirror and microlens have a wide range of applications, including optical pickup [1], multiphoton microscopy [2], Fourier transform spectrometry [3,4], confocal microscopy [5], optical coherence tomography [6], and micro optical diffusion sensing [7,8,9]. MEMS scanners based on electrostatic [2,7,10,11], piezoelectrical [12], and electromagnetic [13] actuation mechanisms can achieve high-speed scanning. For example, Oda et al. [11] reported an electrostatic comb-drive MEMS mirror with a sensing function, which achieved a vertical displacement of 3 µm with approximately 40 V. Chen et al. [12] demonstrated 145 µm out-of-plane actuation with a 2 kHz resonant frequency using symmetrical eight piezoelectric unimorph driving. Compared with electrostatic, piezoelectrical, and electromagnetic actuations, an electrothermal MEMS scanner can achieve large displacement (several hundred micrometers) without resonant operation. To date, various novel designs for electrothermal MEMS scanners with vertical out-of-plane actuation have been proposed [3,4,5,6,8,14,15,16]. For example, Zhang et al. [14] presented a lateral shift-free actuator design using three bimorph hinges and two multimorph segments to compensate for the lateral shift. Their actuator achieved a vertical displacement of 320 µm. Zhou et al. [15] recently reported an electrothermal MEMS mirror with a high reliability and 114 µm scanning using an inverted-series-connected structure, which survived significant long-term operation with little characteristic change.

Kirigami, a variation of origami, is a promising design method for building out-of-plane structures by paper cutting and folding. The design concepts of kirigami and origami have been introduced in the engineering of a large variety of nano-, micro-, and macroscale functional films, such as mechanical materials [17,18,19,20,21,22,23], photonic materials [24,25,26,27], biomedical devices [28,29,30], biomimetic robotics [31], and electronic devices [32,33]. Mechanically-actuated devices particularly require the ability to control the transition between folded and unfolded states. A planer stretchable film is kinematically manipulated with external stretching tethers [20,24]. The responsive film materials provide self-reconfigurable folding and unfolding when exposed to a change in environmental temperature [34,35,36], the addition of a solvent [37,38], or irradiation by lasers [39]. For example, Tolley et al. [35] demonstrated self-folding origami shapes composed of shape memory polymer, which is activated by uniform heating in an oven for less than 4 min. Jamal et al. [37] reported that a differential photo-crosslinked epoxy polymer, SU-8, was reversibly folded and unfolded by de-solvation and re-solvation to develop microfluidic devices that flatten out and curl up. However, these folding and unfolding mechanisms are not applicable to the fast-scanning MEMS actuator that is necessary for electrical control on a microscale. Moreover, electrically responsive film materials compatible with the fabrication of microscale architecture are required.

In this study, we explore the feasibility and potential of a kirigami-inspired electrothermal MEMS scanner that enables large vertical actuation with a high fill factor. Based on the concept of a thermal bimorph being folded and unfolded by the thermal expansion difference induced by Joule heating and natural cooling, the freestanding kirigami film on which bimorphs are placed is electrothermally folded into an out-of-plane structure. In this design, the film material combinations suitable for electrothermal self-reconfigurable folding and unfolding are determined, and the kirigami cuttings and thermal bimorphs are aligned to generate vertical displacement with a high area efficiency. To fabricate the freestanding electrothermal kirigami film with a 2 mm diameter and high fill factor, spontaneous film folding due to residual stress, which determines the initial position, is controlled. Finally, the potential of fast, large-displacement scanning with a high fill factor is experimentally examined.

## 2. Kirigami-Inspired Electrothermal MEMS Scanner

### 2.1. Design

**Figure 1:**
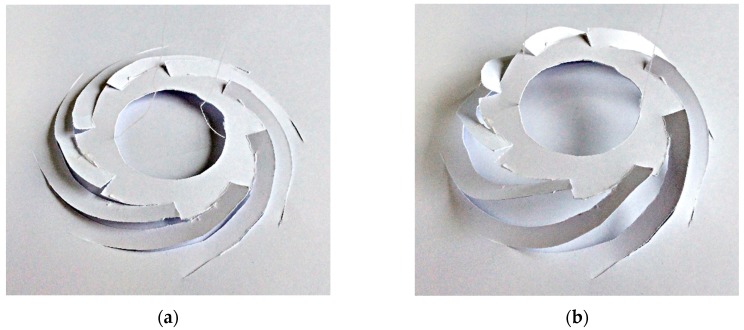
Paper model of the proposed kirigami scanner: (**a**) unfolded state and (**b**) folded state.

**Figure 2:**
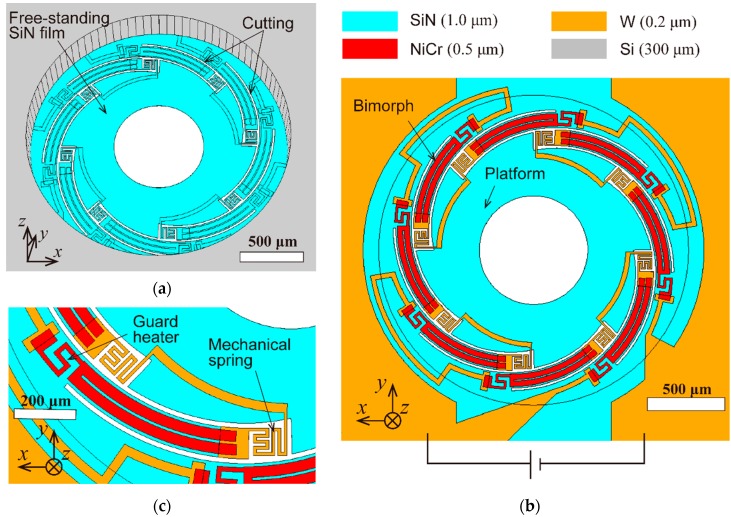
Design of the electrothermal kirigami scanner: (**a**) frontside perspective view of freestanding SiN film (1.0 µm thickness, 2 mm in diameter) on which the spiral-curved cuttings are strategically placed; (**b**) backside view of SiN film where NiCr patterns (0.5 µm thickness) and W patterns (0.2 µm) are deposited; (**c**) backside detailed view of the thermal bimorph beam.

Figure 1 shows a 3D paper model of the proposed scanner. Inspired by the kirigami concept in which a plane paper is transformed into out-of-plane architecture by cutting and folding, the freestanding film was electrothermally folded into an out-of-plane structure. When switching the voltage on or off, the platform for the microlens and micromirror was vertically lifted or lowered. Figure 2 illustrates the design schematic for the electrothermal kirigami MEMS scanner. As Figure 2a shows, the freestanding SiN film on which the spiral-curved cuttings were strategically placed was formed on a Si substrate. The SiN film (1.0 µm thickness) was 2 mm in diameter, and the platform for the micromirror and microlens was 1.3 mm in diameter. The fill factor (i.e., the ratio of the area of the platform to the area of the freestanding kirigami film) was 42%. The extra Si substrate could be removed by the fabrication process to make a small circular chip. As Figure 1b shows, NiCr patterns (0.5 µm thickness) and W patterns (0.2 µm) were deposited on the backside of the SiN film. Figure 2c shows the details of the thermal bimorph beam. The platform was connected to the bimorph with a serpentine-shaped mechanical spring. To suppress heat leakage from the bimorph, the NiCr guard heater was introduced to the bottom area. To suppress the temperature increase at the spring, W patterns, which have a higher electrical conductivity, were deposited on the spring. When voltage was applied to the one-stroke electrical circuit composed of NiCr and W patterns, the all-spiral curved NiCr/SiN bimorph area bent and folded in the vertical direction by Joule heating.

The material properties of the kirigami film are important for reconfigurable electrothermal actuation with large displacement. Film materials with large coefficient of thermal expansion (CTE) differences must be folded so as not to exceed the metal yield strength or fracture strength. Table 1 compares the properties of common materials used in MEMS actuators. SiN was selected as a rigid, freestanding film material with a higher Young’s modulus and yield stress than SiO_2_ or Poly-Si. NiCr and SiN were chosen as the thermal bimorphs. According to Table 1, the yield stress of NiCr is several times higher than that of Cu and Al, while the CTEs of Al and Cu are slightly higher than the CTE of NiCr. The thicknesses of SiN and NiCr are 1 and 0.5 µm, respectively, and were determined by the calculation of cantilever displacement.

### 2.2. Simulation Analysis

**Figure 3:**
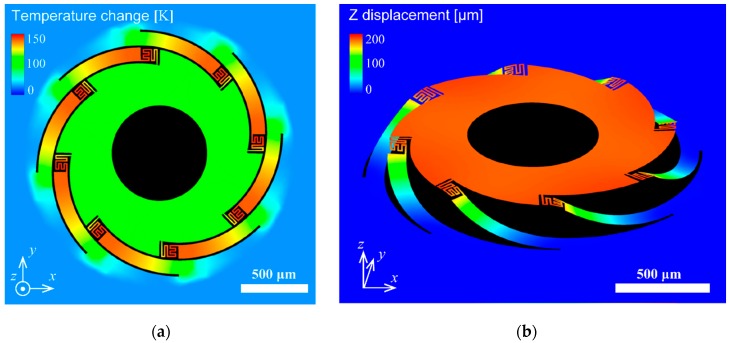
Finite element modeling (FEM) simulation of the microelectromechanical system (MEMS) scanner: (**a**) temperature distribution and (**b**) out-of-plane actuation with approximately 0.2 mm vertical displacement.

**Figure 4:**
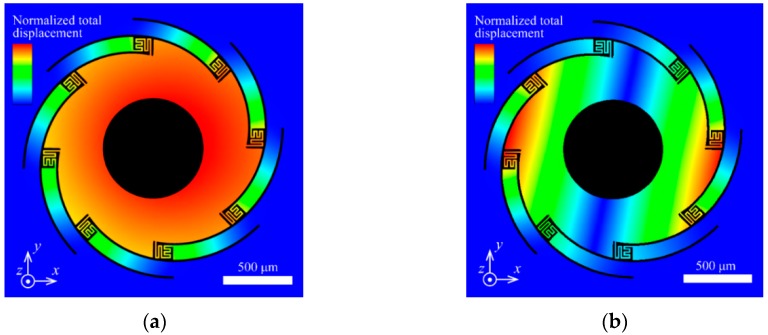
Modal simulation of the MEMS scanner: (**a**) first resonant mode, vertical piston, 1.4 kHz; (**b**) second resonant mode, lateral tilt, 1.8 kHz.

To verify the out-of-plane actuation, 3D models were built, and electro-thermo-mechanical analyses were performed using the CoventorWare© finite element modeling (FEM) tool (Coventor, Inc., Fremont, CA, USA). The thermal conductivity of the freestanding SiN film was particularly considered. In general, the thermal conductivity of the nanoscale thin film was lower than that of the bulk state due to phonon scattering at the interface grain. Figure 3 shows the temperature distribution and vertical actuation using the film value [46]. A selective temperature rise in the bimorph area was observed, and NiCr thermal guard heaters suppressed the temperature decrease in the bimorph bottom area. As seen in Figure 4b, the proposed electrothermal actuator could achieve approximately 0.2 mm displacement in the vertical direction. The resonant vibration modes were simulated, and Figure 4 shows the results. The first mode was piston, and the second mode was tilting. The frequencies were 1.4 and 1.8 kHz, respectively.

## 3. Fabrication

**Figure 5:**
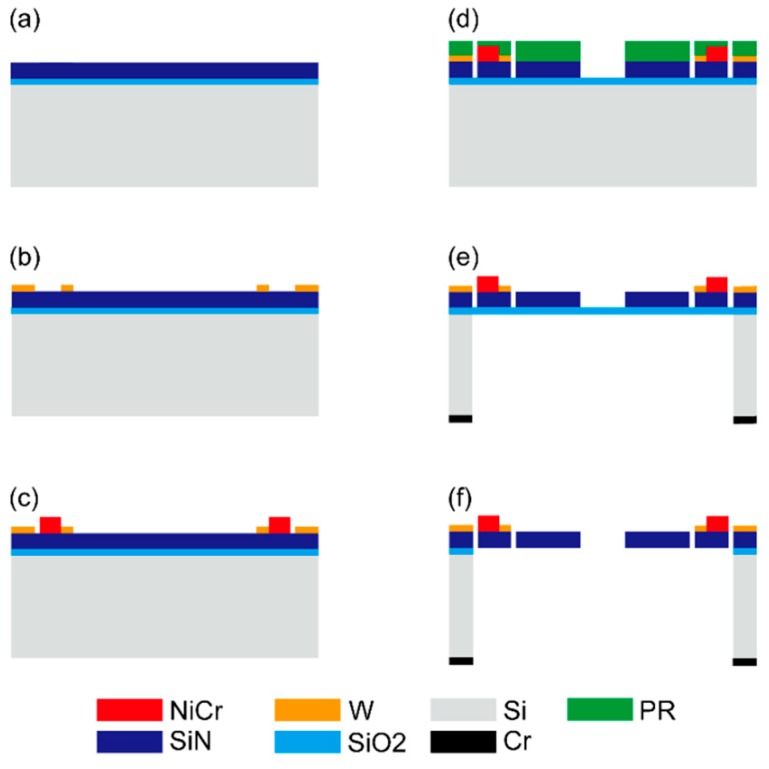
Fabrication flow of the electrothermal kirigami MEMS scanner: (**a**) SiO_2_/SiN film with an approximately 30 MPa compressive strength was deposited by plasma-enhanced chemical vapor deposition (PECVD). (**b**) W patterns were deposited by lift-off processes. (**c**) NiCr films with 180 MPa tensile residual stress were deposited by sputtering and patterned by wet etching. (**d**) After PR patterning, microscale kirigami cuttings were placed on SiN film by reactive-ion etching (RIE). (**e**) After Cr mask patterning and PR removal, the Si substrate was etched by the backside DRIE to form the SiO_2_/SiN film. (**f**) Freestanding SiN kirigami film on which NiCr and W patterns were deposited was formed by SiO_2_ removal with vapor hydrofluoric acid (HF) etching.

**Figure 6:**
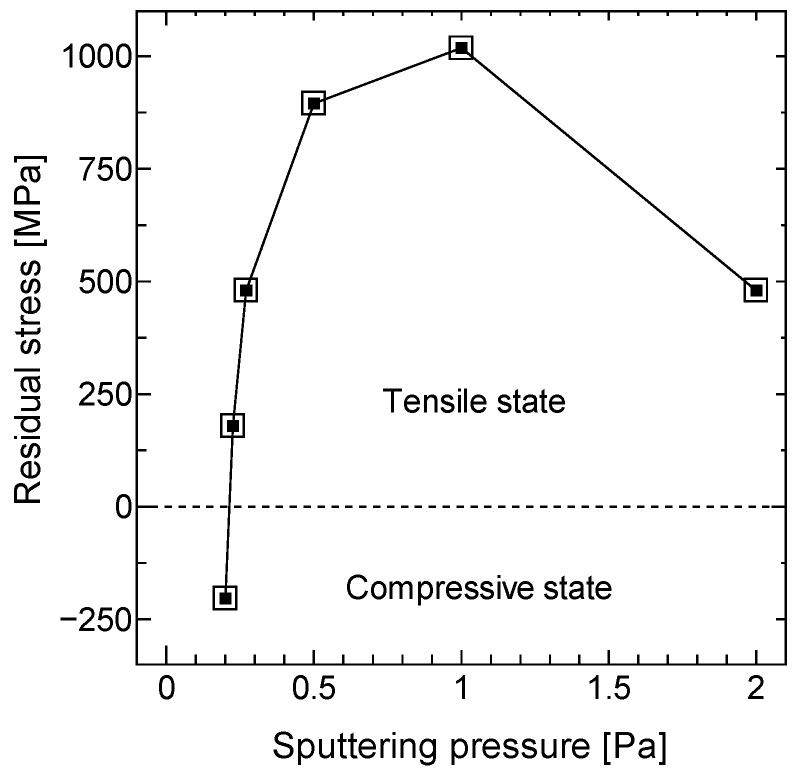
Residual stress of the NiCr film versus Ar sputtering pressure. In this study, the Ar pressure was set to 0.23 Pa for deposition of the NiCr film with 180 MPa tensile stress.

Figure 5 represents the fabrication process flow of the kirigami-film actuator. To fabricate the freestanding SiN film on which NiCr patterns were deposited with a high area efficiency, it was necessary to control the residual stress of both SiN and NiCr. First, an SiO_2_ film with low residual stress was deposited on a single-side polished Si wafer with a 300 µm thickness and 100 mm diameter, as shown in Figure 5a. To prevent the SiN film from shrinking after removal of the SiO_2_ film underneath, the SiO_2_ film with low residual stress, estimated to have a 30 MPa compressive strength, was grown by plasma-enhanced chemical vapor deposition (PECVD) using TEOS. This functioned as an etch stop layer during two processes: (1) SiN film reactive-ion etching to align the kirigami cuttings on the SiN film and (2) Si deep reactive-ion etching (DRIE) to form the freestanding SiN/SiO_2_ film. A low-residual SiN (1.0 μm) film was then deposited by PECVD. To produce a low-stress SiN film, which is composed of alternating tensile and compressive layers, low and high radio frequency (RF) mix fabrication was used [47]. The residual stress of the SiN film was adjusted to a 25 MPa compressive strength, approximately equal to the residual stress of the SiO_2_ film, to further prevent film shrinkage by different residual stresses on the SiO_2_ film. Next, the W pattern was deposited (0.2 μm) by RF magnetron sputtering and a lift-off process, as shown in Figure 5b. NiCr alloy (80% Ni–20% Cr) patterns (0.5 μm) were deposited by RF sputtering and wet etching, as shown in Figure 5c. The residual stress of the NiCr patterns caused the initial curling of the bimorphs, resulting in the initial elevation of the platform. Moreover, the compressive/tensile state determined the direction of the initial elevation. The residual stress of NiCr patterns was controlled by adjusting the sputtering gas pressure. Figure 6 shows the residual stresses of NiCr films (0.5 μm) versus process pressure using fixed sputtering power. The residual stresses were estimated from the curvature of the film-coated substrates using Stoney’s formula [48]. As the sputtering Ar pressure was increased, the sputtered film transitioned from a compressive state to tensile state. After reaching a maximum tensile strength, the stress was decreased with a further increase in the pressure. This tendency, which has also been reported in W films [49] and Ta films [50], can be attributed to the change of the film qualities caused by mean free paths of Ar and NiCr atoms. The NiCr film with 180 MPa in a tensile state was deposited because the tensile stress caused the opposite direction to actuate, which did not result in displacement reduction. After photoresist (PR) masking pattern-inverse kirigami-cutting geometry, the SiN film was etched by reactive-ion etching (RIE) and stopped at the SiO_2_ layer, as shown in Figure 5d. After backside Cr mask patterning by sputtering and wet etching followed by frontside PR removal, the Si substrate was etched by the backside DRIE to form the SiO_2_/SiN film, as shown in Figure 5e. To prevent erosion of the frontside metal pattern by backside Cr etchant, the PR was removed by acetone immersion and O_2_ ashing after Cr pattering. Finally, the SiO_2_ film was removed by vapor hydrofluoric acid (HF) release, and the freestanding kirigami film on which NiCr and W were patterned was formed, as shown in Figure 5f.

**Figure 7:**
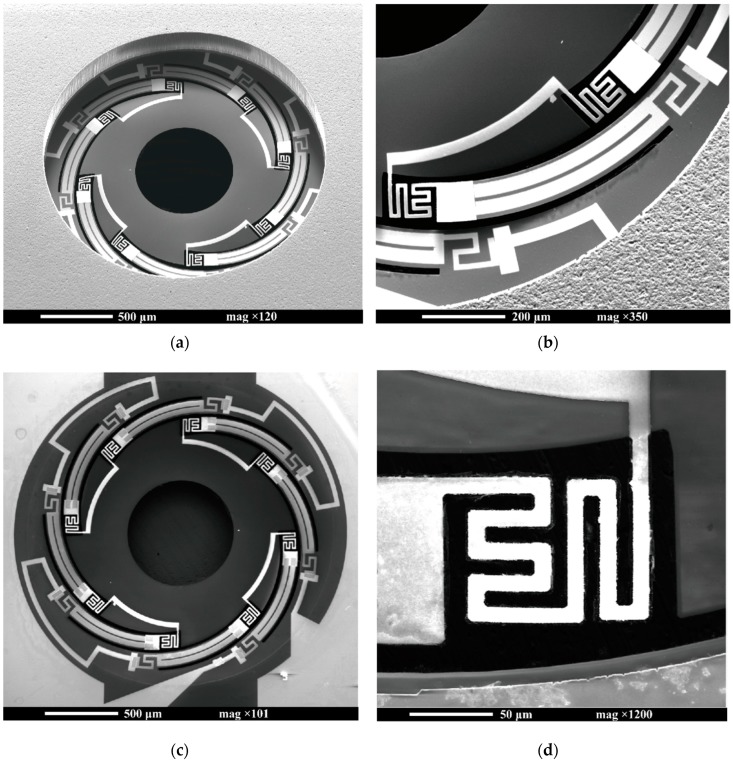
SEM images of the fabricated electrothermal kirigami scanner: (**a**) frontside perspective view of freestanding SiN film (2 mm in diameter) on which the spiral-curved cuttings were strategically placed; (**b**) detailed view of thermal bimorph area, including the serpentine spring and guard heater; (**c**) backside view of the SiN film where NiCr patterns and W patterns were deposited; (**d**) detailed view of the serpentine mechanical spring.

Figure 7 shows the scanning electron microscope (SEM) images of an electrothermal kirigami MEMS scanner. The freestanding SiN film on which the spiral-curved cuttings were strategically placed was formed on the Si substrate, as shown in Figure 7a. No destruction was observed in the bimorph area, including the guard heater and serpentine spring (Figure 7b,d). Initial displacement of the platform was 20 µm above the substrate level. Platform tilting was estimated to be approximately 0.6° by microscope focusing. The one-stroke electrical circuit composed of NiCr and W patterns was successfully deposited on the SiN film (Figure 7c). The measured resistance of the scanners was 4.6 kΩ at room temperature.

## 4. Experimental Tests

### 4.1. Static Response

**Figure 8:**
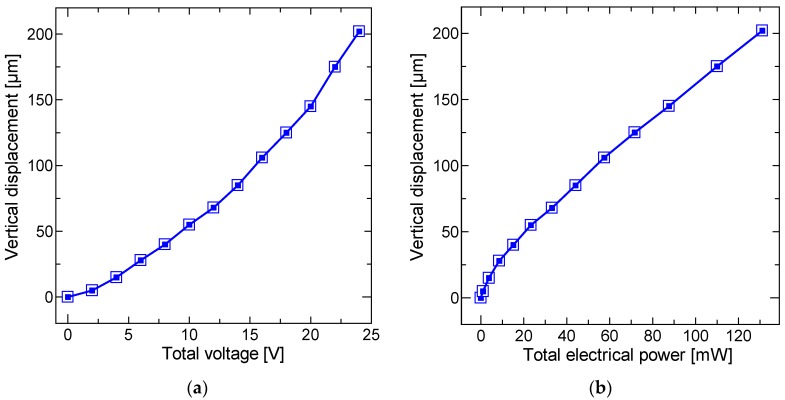
Static response of electrothermal kirigami scanner when the voltage was applied to eight NiCr/SiN bimorphs. The vertical displacement was precisely measured by microscope focusing of a selected point on the platform: (**a**) vertical displacement versus total applied voltage, and (**b**) vertical displacement versus total electrical power.

**Figure 9:**
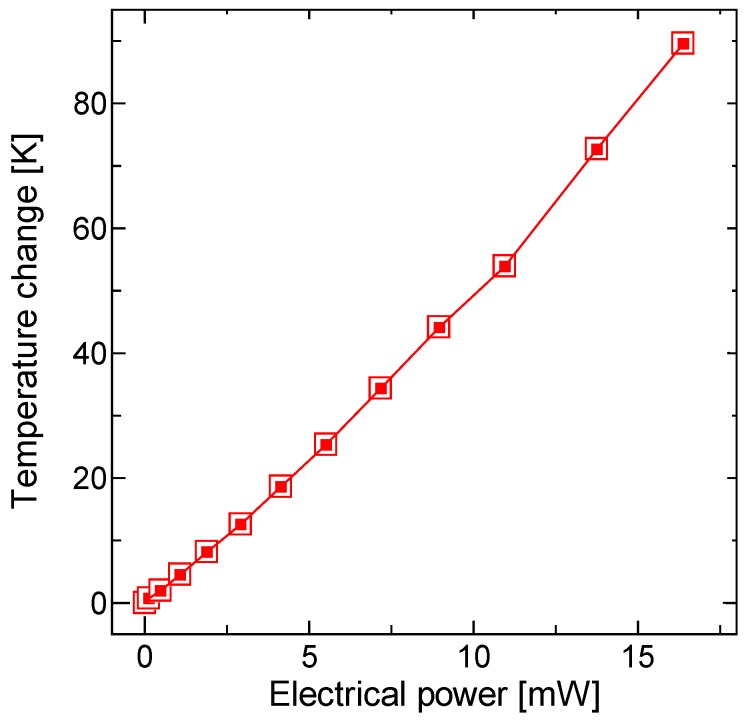
Measured temperature change versus applied electrical power for each bimorph. The temperature rise at the NiCr/SiN bimorph was measured using infrared thermography. The power applied to one bimorph was estimated from the total power.

The direct current (DC) responses of the proposed scanners were characterized. Through contact of the electrical probe and the W electrode patterned on the Si frame, the voltage was applied to the one-stroke circuit composed of W and NiCr patterns that was deposited on the SiN film. The power supplied to the scanner was calculated from the input voltage and the measured current. The vertical displacement was precisely measured by microscope focusing of a selected point on the platform. Figure 8 shows the vertical displacements of the platform versus the applied voltage and power. A vertical displacement of 200 µm was achieved at only 131 mW, as shown in Figure 8b. The temperature rise at the NiCr/SiN bimorph was measured using infrared thermography (TVS-8500, Nippon Avionics, Tokyo, Japan, measurement accuracy of ± 2 °C at T ≤ 373 K). Figure 9 shows the temperature change with respect to the applied electrical power of the single bimorph. The applied power was calculated by dividing the total power by the number of bimorphs. The measurement point was set to the center point of the thermal bimorph. A temperature rise of approximately 90 K was obtained at 200 µm displacement.

### 4.2. Dynamic Response

**Figure 10:**
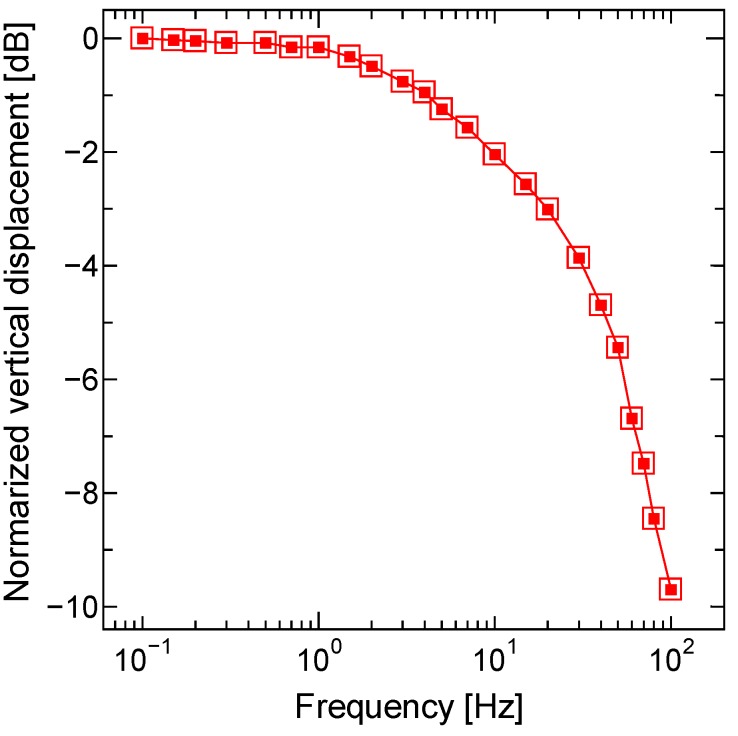
Frequency response of the electrothermal kirigami scanner when applying a sinusoidal wave voltage. The frequency response of the vertical actuation was measured by spot displacement of the beam reflected by the platform.

The dynamic responses in the low-frequency range of the MEMS scanner were characterized. Figure 10 shows the measured frequency response when applying a sinusoidal wave voltage to the single electrical circuit. The frequency response of the vertical actuation was measured by spot displacement of the beam reflected by the platform. The displacement of the beam spot was measured using a position sensitive detector. The applied voltage was (V_o_ + V_o_sin(2π*ft*), V_o_ = 7 V), corresponding to the maximum 85 µm displacement of DC operation, because of the spatial restriction of the optical path for the experiment on the frequency responsivity check. The low-frequency band was mainly determined by the thermal response of the scanner, and the 3 dB cutoff frequency was approximately 20 Hz. The mechanical resonant frequency of the piston mode was estimated to be 1.4 kHz. Therefore, no resonant peak was observed in the low-frequency range.

## 5. Conclusions

Kirigami, a traditional Japanese art of paper cutting and folding, is a promising engineering method for creating out-of-plane structures. This paper proposes a kirigami-inspired electrothermal MEMS scanner that obtains large vertical displacement by out-of-plane film actuation. Film material combinations suitable for electrothermal self-reconfigurable folding and unfolding were selected, and microscale cuttings were strategically placed to generate a large displacement. The freestanding electrothermal kirigami film with a 2 mm diameter and high fill factor was completely fabricated by careful stress control in the microfabrication process. A 200 μm vertical displacement with 131 mW and a 20 Hz responsive frequency was experimentally demonstrated as a unique function of electrothermal kirigami film. The proposed design, fabrication process, and experimental tests validate the proposed scanner’s feasibility and potential for large-displacement scanning with a high fill factor.

## Figures and Tables

**Table 1:** Material selection of the electrothermal kirigami scanner [40,41,42,43,44,45].

Material	Coefficient of Thermal Expansion [10^−6^/K]	Young’s Modulus [GPa]	Yield Strength/Fracture Strength [GPa]
SiN	1.6	252	5.8
Poly-Si	3.0	179	1.1
SiO_2_	0.4	70	0.8
Al	23.6	70	0.2
Cu	16.9	120	0.3
NiCr	14.2	220	2.2
